# Remdesivir: Real-World Effectiveness and Safety in Individuals Hospitalized for Non-COVID Reasons and Non-Hospitalized High-Risk Patients During the Omicron Era in Greece

**DOI:** 10.3390/microorganisms14020441

**Published:** 2026-02-12

**Authors:** Nikos Pantazis, Spyridon Kontos, Evmorfia Pechlivanidou, Nikolaos V. Sipsas, Diamantis Kofteridis, Periklis Panagopoulos, Vassiliki Rapti, Symeon Metallidis, Karolina Akinosoglou, Dimitra Kavatha, Haralampos Milionis, Ioannis Kalomenidis, Ioannis Katsarolis, Vasiliki E. Georgakopoulou, Vasileios Petrakis, Garyfallia Poulakou, Olga Tsachouridou, Markos Marangos, Anastasia Antoniadou, Eleni Polyzou, Eleni Papantoniou, Pinelopi Kazakou, Eirini Christaki, Theofani Rimpa, Sotirios P. Karagiannis, Giota Touloumi

**Affiliations:** 1 Department of Hygiene, Epidemiology and Medical Statistics, Medical School, National & Kapodistrian University of Athens, 11527 Athens, Greece; 2 Infectious Diseases and COVID-19 Unit, General Hospital of Athens Laiko, Medical School, National & Kapodistrian University of Athens, 11527 Athens, Greece; 3 Department of Internal Medicine, University General Hospital of Heraklion, Faculty of Medicine, School of Health Sciences, University of Crete, 71003 Heraklion, Greece; kofterid@med.uoc.gr; 4 Department of Internal Medicine, University General Hospital of Alexandroupolis, Department of Medicine, Democritus University of Thrace, 68100 Alexandroupolis, Greece; 5 3rd Department of Internal Medicine, Athens Hospital for Diseases of the Chest “Sotiria”, Medical School, National & Kapodistrian University of Athens, 11527 Athens, Greece; 6 1st Department of Internal Medicine, AHEPA University General Hospital, Medical School, Aristotle University of Thessaloniki, 54124 Thessaloniki, Greece; 7 Department of Internal Medicine, University General Hospital of Patras, Department of Medicine, University of Patras, 26504 Rio, Greece; 8 4th Department of Internal Medicine, Attikon University General Hospital, Medical School, National & Kapodistrian University of Athens, 26504 Athens, Greece; 9 Department of Internal Medicine, University General Hospital of Ioannina, Faculty of Medicine, Department of Internal Medicine, School of Health Sciences, University of Ioannina, 45110 Ioannina, Greeceeirini.christaki@uoi.gr (E.C.); 10 1st Department of Critical Care & Pulmonary Service, Evangelismos General Hospital, Medical School, National & Kapodistrian University of Athens, 11527 Athens, Greece; ikalom@med.uoa.gr (I.K.);; 11 Medical Affairs, Gilead Sciences Hellas and Cyprus, 17564 Paleo Faliro, Greece; ioannis.katsarolis@gilead.com

**Keywords:** COVID-19, remdesivir, incidentally COVID-19-positive patients, non-hospitalized high-risk COVID-19-positive patients, effectiveness, safety

## Abstract

Remdesivir is recommended for hospitalized patients with severe COVID-19 and for those at high risk of progression. Real-world Omicron-era data on incidental COVID-19 and high-risk outpatients remain limited. We conducted a multicenter retrospective cohort study (ReEs-COVID19) in Greece (June–December 2022) including adults with PCR-confirmed SARS-CoV-2 infection who received remdesivir. Hospitalized patients with incidental COVID-19 (Group A, *n* = 138) and high-risk outpatients (Group B, *n* = 312) were analysed. Outcomes included clinical deterioration, mortality, and adverse events. Group A patients were older with more comorbidities. Remdesivir was initiated earlier in Group A (median 1 vs. 2 days) but with a more heterogeneous duration (48.9% vs. 97.8% in Group B, which received the standard 3-day regimen). Clinical deterioration due to COVID-19 occurred in 5.8% vs. 0.6%, and 30-day mortality was 18.1% (25/138) in Group A, including 10 COVID-19-related deaths (7.2%). Group B had two deaths (0.6%), none COVID-19-related. Adverse events were uncommon, with mild kidney injury in 3.6% of Group A and hepatotoxicity in 2.2% vs. 0.3%. In high-risk outpatients, the ReEs-COVID19 study confirmed the effectiveness and safety of remdesivir’s profile. Among incidental cases, two distinct disease patterns were identified, associated with different remdesivir regimens and highlighting the importance of comorbidities and the need for tailored clinical interventions.

## 1. Introduction

Since the emergence of SARS-CoV-2, the virus causing COVID-19, in late 2019, over 778 million cases and 7 million deaths have been reported globally [1], while swift research advances have led to effective diagnostics, vaccines, and therapies [2].

Remdesivir was the first antiviral agent authorized for the treatment of COVID-19. After promising findings in preclinical and early clinical stages [3,4,5], randomized controlled trials demonstrated remdesivir’s efficacy in shortening recovery time and improving patient outcomes [6,7,8]. Based on these findings, remdesivir received conditional authorization from the European Medicines Agency in July 2020 [9] and was subsequently fully approved by the US Food and Drug Administration in October 2020 [10]. In Greece, remdesivir first became accessible through clinical trials and compassionate-use schemes in early 2020. Following the European Medicines Agency’s authorisation for use in hospitalized patients with severe COVID-19, remdesivir was incorporated almost immediately into the national therapeutic algorithm and rapidly adopted across hospital settings. After publication of the PINETREE trial in late 2021 [11], its indication was expanded in early 2022 to include patients at high risk for severe disease, which was subsequently reflected in national clinical practice.

The ongoing evolution of SARS-CoV-2 has led to the emergence of multiple variants, each exhibiting unique characteristics in terms of transmissibility and disease severity [12]. Overall, the literature to date supports the early use of remdesivir in a broad range of hospitalized and nonhospitalized individuals experiencing COVID-19 at various degrees of severity. Although remdesivir continues to exhibit antiviral activity against multiple SARS-CoV-2 variants [13], evidence regarding its clinical effectiveness in real-world settings during the Omicron era is available, but it is relatively limited for outpatient or atypical cases [14,15,16].

In high-comorbidity hospitalized patients, COVID-19 severity is influenced not only by viral pathogenicity but also by host-related factors such as frailty, impaired immune responses, and heightened vulnerability to secondary or nosocomial infections. In such individuals, even mild SARS-CoV-2 infection may contribute to clinical deterioration, although the extent to which early antiviral therapy provides benefit in this context remains uncertain.

Data on the use of remdesivir in patients with incidental SARS-CoV-2 infection, i.e., those hospitalized for unrelated conditions but found to be positive for SARS-CoV-2 infection during their hospitalization, remain scarce [17]. Similarly, although the PINETREE trial demonstrated the efficacy of early remdesivir use in high-risk outpatients [11], evidence from routine clinical care in this population during the Omicron period is limited to retrospective studies, with heterogeneous inclusion criteria and outcomes [18,19]. Further investigation is warranted to inform treatment strategies for these underrepresented yet clinically important patient groups.

To address this gap, the ReEs-COVID19 study was initiated to collect real-world data on COVID-19 patients treated with remdesivir in Greece. Phase 1 focused on hospitalized patients during the pre-Omicron period and has already yielded valuable insights into remdesivir use and outcomes [20]. In its current second phase, the study has been extended to the Omicron era and now includes, in addition to patients hospitalized for COVID-19, (a) patients hospitalized for non-COVID-19 reasons who were incidentally diagnosed with SARS-CoV-2 infection during their hospitalization and received remdesivir during hospitalization (“Incidental COVID-19”; Group A) and (b) high-risk, non-hospitalized outpatients who received early remdesivir treatment to avoid progression to severe COVID-19 (“High-risk outpatients”; Group B).

The present analysis aims to describe and compare the “Incidental COVID-19” and “High-risk outpatients” groups in terms of clinical progression, rehospitalization, mortality, safety outcomes and remdesivir treatment patterns.

## 2. Methods

### 2.1. Study Design and Eligibility Criteria

ReEs-COVID19 is a retrospective observational cohort study of adults (≥18 years old) with PCR-confirmed SARS-CoV-2 infection who received remdesivir during the Omicron variant era (1 June 2022 to 31 December 2022) in one of the eight participating hospitals across Greece, identified through hospital pharmacy records. The current analysis focuses on (a) Incidental COVID-19 cases (group A): patients hospitalized for reasons unrelated to COVID-19 who were incidentally found to be SARS-CoV-2-positive during hospitalization and received remdesivir as prophylaxis based on their high-risk profile and (b) High-risk outpatients (group B): patients with mild or moderate COVID-19 who were considered at high risk for progression to severe disease and received early remdesivir treatment. Due to national regulations requiring intravenous therapy to be administered in a hospital setting, all patients in group B had short-stay admissions (e.g., day clinic or brief hospitalization) for remdesivir infusion.

Patients admitted directly to the intensive care unit (ICU) on the first day of hospitalization or enrolled in clinical trials for other COVID-19 treatments were excluded.

Data were collected from patient files and hospital records, including demographic and social characteristics, clinical status at presentation, vaccination history, comorbidities, COVID-19 symptom onset and diagnosis dates, prior COVID-19 episodes, and laboratory findings. Remdesivir treatment details and clinical outcomes for up to 30 days after hospital admission (group A) or the start of remdesivir treatment (group B) were recorded. Follow-up phone calls were conducted to capture post-discharge events not documented in hospital records.

### 2.2. Endpoints

The primary endpoints were (a) clinical deterioration defined as the new initiation of supplemental oxygen or an increase in oxygen requirements attributable to COVID-19, (b) rehospitalization for COVID-19 within 30 days after discharge (group A) or hospitalization for COVID-19 within 30 days after completion of remdesivir treatment (group B) and (c) death from any cause within 30 days after hospital admission (group A) or remdesivir initiation (group B).

Secondary endpoints included (a) occurrence of hepatic and renal laboratory abnormalities and (b) patterns of remdesivir use, including timing from admission (group A only) or diagnosis to treatment initiation and total duration of therapy.

Liver function abnormalities were classified as mild/moderate if ALT and/or AST levels were between 3 and 5 times the upper limit of normal (ULN) or severe if ALT and/or AST levels were above 5× ULN.

Renal function abnormalities were categorized using the three-stage classification proposed by the “Kidney Disease: Improving Global Outcomes” (KDIGO) criteria for acute kidney injury (AKI) [21]. Any hepatic or renal event occurring during remdesivir administration or within two days after the final dose was considered a potential remdesivir-associated adverse event, based on known remdesivir pharmacokinetics.

### 2.3. Informed Consent

No study-specific informed consent was required due to the retrospective design of this study and the use of anonymized data. All patients had previously provided written general informed consent at their treating hospitals, permitting the use of anonymized clinical data for research purposes in accordance with applicable regulations.

### 2.4. Statistical Analysis

Baseline characteristics were summarized using median and interquartile ranges (IQR) for continuous variables and absolute (N) and relative (%) frequencies for categorical ones. Between-group differences were assessed using Mann-Whitney U-tests for continuous variables and Fisher’s exact tests for categorical ones. Statistical analysis was performed using Stata (StataCorp. 2023. Stata Statistical Software: Release 18. College Station, TX, USA: StataCorp LLC.).

## 3. Results

### 3.1. Patients’ Characteristics

This study included a total of 450 patients, with 138 of them belonging to Group A (Incidental COVID-19 cases) and 312 in Group B (High-risk outpatients). Their characteristics are summarized in Table 1. Median (IQR) age was similar in groups B and C (70, 54–82 vs. 68, 58–76 years, respectively), but with substantially more patients in the 80+ years category in Group A (32.6% vs. 17.6% in Group B). Females represented 45.8% of the total study population, with no significant difference between groups. Most patients resided in urban areas (85.1%) and were of Greek nationality (94.7%), with no significant differences between the two groups.

The median (IQR) Charlson Comorbidity Index was significantly higher in Group A (4, 3–5) compared to Group B (3, 2–5). More patients in Group A had two or more comorbidities (72.5% vs. 60.9%). Cardiovascular disease (i.e., presence of at least one of atrial fibrillation, myocardial infarction, heart failure, hypertension, stroke, dyslipidaemia, or coronary heart disease) was significantly more prevalent in Group A (80.4% vs. 67.6%). Other significantly more frequent comorbidities in Group A included chronic kidney disease (26.1% vs. 10.3%), hypertension (62.3% vs. 45.8%), dyslipidaemia (48.6% vs. 32.1%), and non-HIV immunosuppression (22.5% vs. 13.8%), while obesity was more common in Group B (6.4% vs. 1.4%). Chronic liver disease was rare but seen only in Group A (1.4%). Other conditions, such as asthma, active cancer, COPD, diabetes, atrial fibrillation, coronary artery disease, heart failure, myocardial infarction, stroke, thyroid dysfunction, obstructive sleep apnoea, and idiopathic pulmonary fibrosis, showed no significant differences.

Analysis of the comorbidity patterns revealed two common patterns among the patients. The first one was dominated by cardiovascular diseases, where hypertension, dyslipidaemia, and coronary artery disease frequently co-occurred, especially in Group A. The second most common pattern involved chronic kidney disease combined with diabetes and hypertension. These patterns align with the higher Charlson Comorbidity Index scores and increased disease severity observed mainly in Group A. In contrast, Group B patients had fewer and less severe comorbidities, with a lower prevalence of chronic kidney disease and cardiovascular conditions. As mentioned earlier, a substantial proportion of patients exhibited non-HIV immunosuppression, with many of them receiving immunosuppressive therapies or having underlying conditions that affect immune function. Active cancer was present in 14.5% of Group A and 18.6% of Group B patients. There was considerable overlap between immunosuppression and active cancer across both groups.

In Group A, the most common reasons for admission included chronic kidney pathology (24.6%), other infections (18.1%), lung pathology (10.9%), biliary pathology (8.0%), cancer (6.5%), and stroke (4.3%), with smaller proportions admitted for myelodysplastic syndrome (1.4%) or other reasons (15.2%) such as anaemia, metabolic disturbances, nonspecific symptoms (e.g., abdominal pain, dizziness), musculoskeletal complaints or minor injuries.

Vaccination coverage was similarly high in both groups, with 83.3% of patients in Group A and 82.7% in Group B being vaccinated at least once (Table 2). The proportion of fully vaccinated patients (defined as those who received at least two doses plus one booster) was 73.9% in Group A and 68.3% in Group B. Regarding recent immunity, 9.4% of Group A and 12.2% of Group B had either vaccination or hospitalization for COVID-19 within the last 4 months, while 21.7% of Group A and 19.9% of Group B patients had vaccination or hospitalization in the last 6 months.

### 3.2. Remdesivir Patterns of Use

The duration of remdesivir treatment (Table 3) varied significantly between groups. The majority (97.8%) of high-risk outpatients (Group B) received a 3-day treatment course, in line with current guidelines. In this group, one patient received remdesivir for only 1 day and two for 2 days, while four patients were treated for longer than the typical 3-day course (two for 4 days and two for 5 days). In contrast, treatment durations were more heterogeneous in incidental COVID-19 cases (Group A): 49.3% received remdesivir for 3 days, 42.8% for 5 days, and smaller proportions for 2 days (0.7%), 4 days (5.1%), 6 days (1.5%), or 7 days (0.7%).

**Figure 1:**
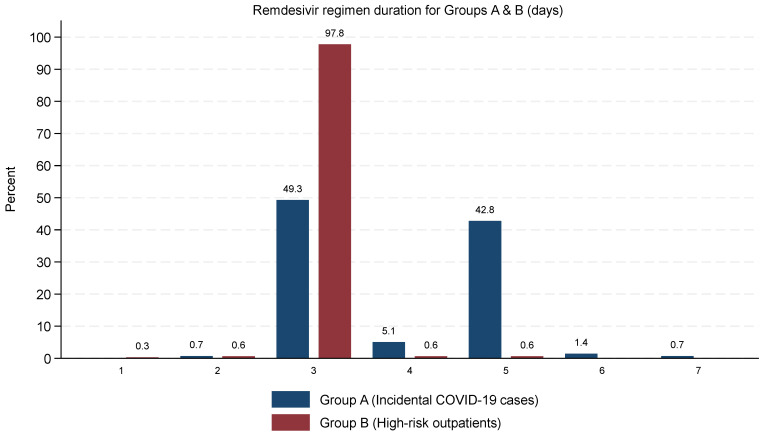
Distribution of remdesivir regimen duration in Incidental COVID-19 cases (group A) and High-risk outpatients (group B).

The subgroup of Group A patients who received an extended course of remdesivir (>3 days; *n* = 69) exhibited markedly worse baseline status compared with those who received treatment for up to 3 days (*n* = 69). Oxygen requirement at presentation (unrelated to COVID-19) was substantially more common in the extended-duration group (29.0% vs. 5.8%), and abnormal chest X-ray findings were also more frequent (43.5% vs. 7.2%). Comorbidities associated with increased vulnerability were more prevalent among patients receiving >3 days of therapy, including immunosuppression (44.9% vs. 4.3%) and COPD (20.3% vs. 7.2%). In addition, a higher proportion of patients in the extended-duration group were aged ≥65 years (81.2% vs. 47.8%). The distribution of remdesivir regimens’ duration in groups A and B is depicted graphically in Figure 1.

The median time between hospital admission of incidental COVID-19 cases (group A) and remdesivir initiation was 4 days (IQR 0–7). The median (IQR) interval between the first positive SARS-CoV-2 test and initiation of remdesivir was significantly shorter in Group A patients (1; 0–1 days) compared to those in Group B (2; 1–3 days).

### 3.3. Clinical Progression and Mortality

**Figure 2:**
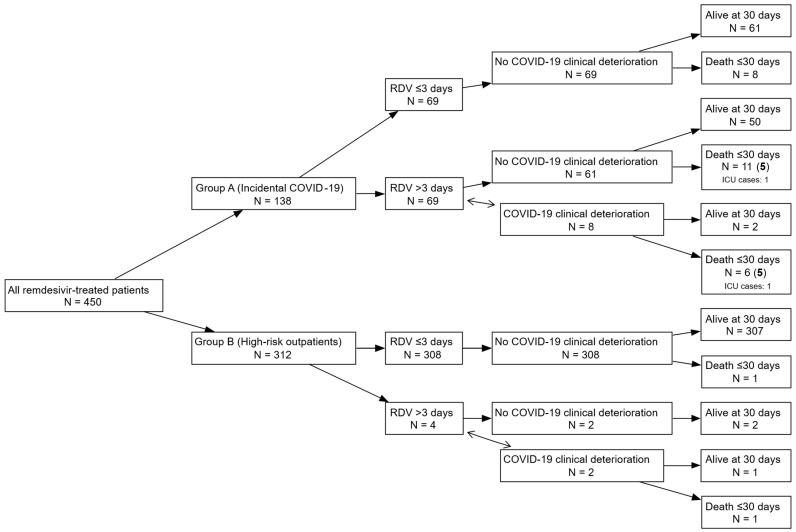
Flowchart of patient groups, remdesivir treatment duration, clinical deterioration due to COVID-19, and 30-day outcomes. Numbers in parentheses after the numbers of deaths indicate those attributed to COVID-19 according to the physicians’ judgment.

Clinical deterioration (Table 4) attributed to COVID-19 was significantly more frequent among patients in Group A compared to Group B. Specifically, 8 (5.8%) patients in Group A experienced a worsening of their initial clinical status, which was attributed to COVID-19 according to the physicians’ judgment, while only 4 (1.3%) of patients in Group B required extension of their 3-day remdesivir regimen. All affected patients with worsening symptoms in both groups received extended remdesivir treatment (5 days). Among these 8 patients in Group A, 6 died within 30 days of admission, with physicians attributing death to COVID-19 in 5 of those cases. Of the two patients in Group B who experienced deterioration, one died, though the death was not considered COVID-19-related (Figure 2).

Progression to critical illness requiring admission to the intensive care unit (ICU) or mechanical ventilation was rare and occurred exclusively in Group A. Two patients (1.4%) in this group required admission to the ICU, both of whom were also intubated. No such events were recorded in Group B.

Thirty-day mortality differed markedly between the two groups. In Group A, 25 patients (18.1%) died within 30 days of admission, of whom 10 deaths were attributed to COVID-19, corresponding to a COVID-19-related mortality of 7.2% of the group. In Group B, two patients (0.6%) died within 30 days of remdesivir initiation, and none of these deaths were attributed to COVID-19. The absolute risk difference for 30-day mortality between Group A and Group B is 17.5% (95% CI: 10.3–24.6%). When extending follow-up to 30 days post-discharge, an additional 7 deaths were recorded in Group A, all unrelated to COVID-19, bringing total mortality in that group to 23.2%.

New hospitalizations within 30 days of discharge or treatment completion were infrequent overall, occurring exclusively in patients of group B (*n* = 3, 1.0%). The causes were unrelated to COVID-19 for all 3 patients.

### 3.4. Renal and Liver Adverse Events

Adverse events were infrequent in both groups (Table 5). Acute kidney injury (AKI) occurred in 3.6% of Group A patients (5/138), exclusively more than two days after completion of remdesivir therapy. No AKI events were recorded in Group B patients after remdesivir administration, but one patient was diagnosed with AKI at baseline. All AKI cases reported were stage 1, indicating mild injury. Liver enzyme elevations associated with remdesivir were rare: 2.2% (3/138) of Group A and 0.3% (1/312) of Group B patients had elevated liver enzymes during treatment, with no significant difference between groups. Additional liver enzyme elevations prior to or on the day of treatment initiation were seen in 0.7% of Group A and 0.3% of Group B. No cases of hepatotoxicity were reported after treatment completion.

## 4. Discussion

In this multicentre, real-world study conducted during the Omicron era in Greece, we evaluated the effectiveness and safety of remdesivir in two insufficiently studied populations: patients hospitalized for causes unrelated to COVID-19 who were incidentally diagnosed with SARS-CoV-2 infection and high-risk outpatients with mild or moderate COVID-19. Our findings indicate that remdesivir use was associated with a low incidence of adverse events in both groups and very low rates of clinical deterioration and mortality in high-risk outpatients.

However, the two study groups showed marked differences in disease progression and mortality, most likely reflecting differences in comorbidity burden rather than treatment timing or duration. Among high-risk outpatients who received remdesivir as early therapy to prevent progression to severe disease, clinical deterioration and COVID-19-related hospitalization were rare, while the 30-day mortality rate was exceptionally low (0.6%). In contrast, patients hospitalized for non-COVID-19 conditions with incidental SARS-CoV-2 infection had significantly higher rates of clinical deterioration (5.8%) and 30-day mortality (18.1%). Only about one-third of these deaths were directly attributed to COVID-19 by the treating physicians, suggesting that excess mortality was primarily driven by advanced age and the higher prevalence of chronic cardiovascular and renal comorbidities in this group.

The two study groups also differed in patterns and timing of remdesivir administration. Among high-risk outpatients, nearly all received the 3-day regimen as recommended for early treatment of mild to moderate COVID-19 by national and international therapeutic guidelines. In contrast, treatment duration among patients with incidental infection was considerably more heterogeneous, with roughly equal proportions receiving 3-day and 5-day regimens. This variability underscores the complexity of inpatient decision-making, where remdesivir administration may be extended in response to evolving symptoms or the presence of serious comorbidities. The median time from diagnosis to remdesivir initiation was shorter in the incidental COVID-19 group (1 day vs. 2 days in outpatients), likely reflecting the timely response of physicians once SARS-CoV-2 infection was identified.

Taken together, these observations suggest that differences in remdesivir timing and duration across the two groups were partly driven by baseline clinical severity and comorbidity burden. This reinforces the need for individualized clinical assessment and tailored therapeutic strategies when managing heterogeneous high-risk populations, particularly among patients hospitalized for non–COVID-19 causes with incidental SARS-CoV-2 infection.

Finally, in our study, remdesivir was well tolerated, with a low frequency of mild hepatic enzyme elevations and no severe hepatotoxicity or treatment-related renal impairment. The few cases of mild acute kidney injury in group A (incidental cases) occurred more than two days after treatment completion and were thus likely unrelated to remdesivir.

Overall, our high-risk outpatient results are generally consistent with those from previous studies evaluating remdesivir in similar patient populations. In the PINETREE trial [11], 2 of 279 participants (0.7%) in the remdesivir arm were hospitalized for COVID-19–related complications within 28 days, with no deaths reported. In the real-world study by Molina et al. [18], conducted during the Omicron era and including 1252 high-risk outpatients, the 28-day all-cause hospitalization rate was 1.3% and mortality was 0.1%. Similarly, in the study by Ramos-Rincón et al. [19], among 211 non-hospitalized high-risk patients treated with a 3-day course of remdesivir, 14 (6.6%) were hospitalized—of which 6 (2.8%) were for COVID-19 progression—and no deaths were attributed to COVID-19. In our Group B, 3 of 312 patients (1.0%) were hospitalized within 30 days after treatment (for reasons unrelated to COVID-19) and 2 deaths (0.6%) occurred, both unrelated to COVID-19. Despite differences in study design, population characteristics, circulating variants, and vaccination coverage, hospitalization and mortality rates were of the same low order of magnitude across these outpatient cohorts.

These findings are consistent with the attenuated clinical severity observed during the Omicron period and the high level of population immunity, as discussed above. Similar observations have been reported in large population-based studies, where the Omicron variant was associated with substantially reduced risks of hospitalization and death compared with Delta, particularly among vaccinated individuals [22,23]. These factors, along with the early initiation of antiviral therapy, likely contributed to the low rates observed in our Group B. Beyond comorbidity burden, host-related molecular factors may also contribute to variability in COVID-19 outcomes. Epigenetic mechanisms and related biomarkers have been proposed as tools to better characterize differences in host immune responses and risk profiles [24]. In parallel, variations in viral entry mechanisms may influence disease presentation and severity [25,26]. Although these mechanisms were not examined in the present study, they provide a biological context for the heterogeneous clinical trajectories observed in real-world high-risk populations.

Remdesivir-related adverse events were uncommon across all studies. In PINETREE [11], any adverse events were reported in 42.3% of participants in the remdesivir arm and 46.3% in the placebo arm, but adverse events considered to be related to the trial regimen occurred in only 12.2% and 8.8%, respectively. Ramos-Rincón et al. [19] noted two moderate adverse events in 211 patients, including one case of gastrointestinal intolerance leading to treatment discontinuation. In our Group B, only one of 312 patients (0.3%) developed mild elevations in liver enzymes during treatment, and no cases of acute kidney injury were recorded after remdesivir administration. These findings confirm the reassuring safety profile of short-course remdesivir in the outpatient setting.

Compared with high-risk outpatients, data on patients hospitalized for non–COVID-19 causes who are incidentally found to have SARS-CoV-2 infection remain limited. Nonetheless, a few studies have recently examined this population. In a Canadian study [17], among 14,290 hospitalized SARS-CoV-2–positive patients, 26% were classified as incidental infections overall. In-hospital mortality was 9% for incidental cases and 17% for those admitted primarily for COVID-19. In a study performed during the Omicron period in Florida, USA [27], in-hospital mortality was 0.6% among patients with incidental infection compared with 15.6% for primary COVID-19 and 8.5% for COVID-related extrapulmonary conditions. In another study from British Columbia [28], 48% of SARS-CoV-2–positive hospitalizations were categorized as incidental, with in-hospital mortality of 6.4% in the incidental group and approximately 13% for those admitted for COVID-19.

In our Group A, overall 30-day mortality following hospital admission was higher than in these studies (25/138, 18.1%), but COVID-19 was considered the primary cause of death in only 9 of these 25 cases. Several factors may account for the higher all-cause mortality observed in our Group A compared with these studies. Patients in our cohort were older (one-third aged ≥80 years) and had a high comorbidity burden (median Charlson Comorbidity Index 4), with a particularly high prevalence of cardiovascular disease (80%), hypertension (62%), dyslipidaemia (49%), and chronic kidney disease (26%). By contrast, incidental SARS-CoV-2 infections reported in the Canadian, U.S., and British Columbia studies generally involved younger and less comorbid populations. Furthermore, whereas those studies reported in-hospital mortality only, our analysis included deaths occurring up to 30 days after discharge, which may further explain the higher overall proportion of deaths despite a relatively small fraction being directly attributed to COVID-19.

Across the aforementioned studies, mortality among patients admitted primarily for COVID-19 was consistently higher than among those with incidental SARS-CoV-2 infection, ranging from 13% to 17% in the for-COVID groups compared with 0.6% to 9% in incidental cases. In contrast, in a recently published study [29], which included patients hospitalized for COVID-19 during the same Omicron period as those in our current Group A, 30-day all-cause mortality was 9.1%, substantially lower compared to that observed in our incidental group (18.1%). Despite a similar age distribution between the two cohorts, the for-COVID group had markedly lower frequencies of serious comorbidities, including cardiovascular disease (58% vs. 80%), chronic kidney disease (6% vs. 26%), and two or more comorbidities (65% vs. 73%). These differences suggest that the higher mortality among patients with incidental infection in our study was driven largely by their greater burden of chronic cardiorenal disease and multimorbidity, rather than by COVID-19 itself. Nonetheless, we cannot exclude the possibility that in some patients the primary admission diagnoses (e.g., kidney/lung pathology, stroke or other infections) may have reflected clinical deterioration partly exacerbated by unrecognised SARS-CoV-2 infection. Such underdiagnosis could have contributed to the severity of presentation and may have acted synergistically with the substantial comorbidity burden to increase mortality risk in this group.

Within the incidental COVID-19 cohort, the subgroup receiving extended remdesivir courses (>3 days) represented a clinically distinct population, characterized by substantially worse baseline status compared with those treated for ≤3 days. All COVID-19 clinical deterioration events and all COVID-19-related deaths within Group A occurred exclusively in this subgroup, aligning with prior evidence that underlying clinical severity is a major determinant of outcomes among remdesivir-treated patients. In comparison to the publication of the severe COVID-19 cohort for the same time period [29], group A mortality was worse both in crude mortality rates (18.1% for group A vs. 9.1% for the severe COVID-19 cohort) and COVID-19 attributable mortality rates (7.2% vs. 0.82%). This further highlights the complexity of incidental COVID-19 cases and the need for tailored interventions. In a randomized trial by Spinner et al. [7], differences in patient trajectories appeared to reflect baseline disease burden rather than treatment duration, while real-world data from the Omicron era [17] similarly highlight the dominant role of comorbidities and physiological vulnerability in shaping clinical outcomes. In this context, the longer remdesivir regimens used in a subset of our incidental cases likely reflect clinicians’ responses to more severe underlying illness rather than progression attributable to SARS-CoV-2 infection itself.

This study has several strengths. It provides real-world evidence on two insufficiently studied patient populations: high-risk outpatients and patients hospitalized for non-COVID-19 causes with incidental SARS-CoV-2 infection, treated with remdesivir during the Omicron period, when disease severity was generally lower and vaccination coverage high. The multicentre design, with several collaborating hospitals from different geographic regions of Greece, together with standardized data collection and inclusion of all eligible cases, enhances the representativeness and reliability of the findings. The use of uniform definitions and endpoints across both groups enables consistent comparisons within and between care settings.

However, certain limitations should be acknowledged. The retrospective observational design precludes causal inference, and the lack of untreated comparators prevents estimation of absolute treatment effects; therefore, the findings should be interpreted as descriptive of routine clinical practice use, safety, and clinical outcomes in high-risk populations. Classification of deaths as COVID-19-related was based on clinical judgment and may be subject to misclassification. Post-discharge outcomes were partly ascertained through follow-up telephone contact, which may have resulted in underreporting of events, particularly those managed outside hospital settings. We also lacked standardized baseline severity indices (e.g., SOFA or frailty scales) and detailed information on functional status and other concurrent treatment regimens. Information on the exact time from symptom onset to diagnosis was not consistently available, which may have limited precise assessment of the timeliness of remdesivir initiation. Although a Charlson Comorbidity Index was available, it does not fully capture acute physiological severity or frailty. Additionally, the possibility of nosocomial SARS-CoV-2 acquisition and variation in viral exposure intensity could not be assessed, and these unmeasured factors may have influenced disease severity and clinical outcomes. While vaccination history was comprehensively documented, information on previous SARS-CoV-2 infections was limited to prior COVID-19-related hospitalizations, which prevented full assessment of infection-induced or hybrid immunity. Although all patients were included during the Omicron-dominant period, systematic identification of SARS-CoV-2 subvariants was not available, precluding stratified analyses by specific Omicron subvariants. Despite these limitations, the study adds valuable evidence on the effectiveness and safety of remdesivir in routine clinical practice and in patient populations that have been underrepresented in clinical trials.

In summary, this multicentre, real-world study provides additional evidence supporting the favourable safety and effectiveness profile of remdesivir across two distinct and understudied populations during the Omicron period. Among high-risk outpatients, early remdesivir administration was associated with very low rates of COVID-19-related hospitalization and death, confirming its preventive role against disease progression. In patients hospitalized for non-COVID-19 causes with incidental SARS-CoV-2 infection, remdesivir was well tolerated and appeared safe, although mortality remained high, most likely due to underlying comorbidities rather than COVID-19 itself. These findings highlight the importance of individualized clinical assessment and early antiviral treatment in high-risk settings, while underscoring the need for prospective studies to better define optimal treatment duration and the role of antivirals in older, multimorbid, or immunocompromised patients.

## Figures and Tables

**Table 1:** Basic demographics, characteristics and hospitalization reasons. All figures are median (IQR) or N (%).

	Group A (Incidental COVID-19 Cases) (*N* = 138)	Group B (High-Risk Outpatients) (*N* = 312)	Total (*N* = 450)	*p* Value
Age (years)	70 (54, 82)	68 (58, 76)	69 (57, 78)	0.167
Age category				0.001
- 18–39	9 (6.5%)	21 (6.7%)	30 (6.7%)	
- 40–59	35 (25.4%)	65 (20.8%)	100 (22.2%)	
- 60–69	24 (17.4%)	83 (26.6%)	107 (23.8%)	
- 70–79	25 (18.1%)	88 (28.2%)	113 (25.1%)	
- 80+	45 (32.6%)	55 (17.6%)	100 (22.2%)	
Gender at birth—Female	64 (46.4%)	142 (45.5%)	206 (45.8%)	0.918
Urbanity				0.803
- Rural	3 (2.2%)	7 (2.2%)	10 (2.2%)	
- Semi-urban	15 (10.9%)	42 (13.5%)	57 (12.7%)	
- Urban	120 (87.0%)	263 (84.3%)	383 (85.1%)	
Greek nationality	130 (94.2%)	296 (94.9%)	426 (94.7%)	0.821
Alcohol Abuse	4 (2.9%)	1 (0.3%)	5 (1.1%)	0.033
Smoking habits				0.013
- Unknown	75 (54.3%)	118 (37.8%)	193 (42.9%)	
- Current smoker	16 (11.6%)	48 (15.4%)	64 (14.2%)	
- Never smoked	29 (21.0%)	99 (31.7%)	128 (28.4%)	
- Former smoker	18 (13.0%)	47 (15.1%)	65 (14.4%)	
Reason for admission (Group A only)				
- Cancer	9 (6.5%)	-	-	-
- Myelodysplastic syndrome	2 (1.4%)	-	-	-
- Stroke	6 (4.3%)	-	-	-
- Lung pathology	15 (10.9%)	-	-	-
- Biliary pathology	11 (8.0%)	-	-	-
- Kidney pathology	34 (24.6%)	-	-	-
- Other	21 (15.2%)	-	-	-
- Other Infection	25 (18.1%)	-	-	-
- Unknown	15 (10.9%)	-	-	-
Charlson Comorbidity Index	4 (3, 5)	3 (2, 5)	4 (2, 5)	0.044
Number of Comorbidities				0.003
- None	6 (4.3%)	48 (15.4%)	54 (12.0%)	
- One	32 (23.2%)	74 (23.7%)	106 (23.6%)	
- Two or More	100 (72.5%)	190 (60.9%)	290 (64.4%)	
Cancer (active disease)	20 (14.5%)	58 (18.6%)	78 (17.3%)	0.345
Obesity	2 (1.4%)	20 (6.4%)	22 (4.9%)	0.030
Immunosuppression (not HIV-related)	31 (22.5%)	43 (13.8%)	74 (16.4%)	0.027
Dyslipidemia	67 (48.6%)	100 (32.1%)	167 (37.1%)	0.001
Hypertension	86 (62.3%)	143 (45.8%)	229 (50.9%)	0.001
Cardiovascular disease	111 (80.4%)	211 (67.6%)	322 (71.6%)	0.006
Chronic kidney disease	36 (26.1%)	32 (10.3%)	68 (15.1%)	<0.001
Chronic liver disease	2 (1.4%)	0 (0.0%)	2 (0.4%)	0.094

**Table 2:** Vaccination and Immunity Status.

	Group A (Incidental COVID-19 Cases) (N = 138)	Group B (High-Risk Outpatients) (N = 312)	Total (N = 450)	*p* Value
Vaccination or hospitalization for COVID-19 in the last 4 months	13 (9.4%)	38 (12.2%)	51 (11.3%)	0.425
Vaccination or hospitalization for COVID-19 in the last 6 months	30 (21.7%)	62 (19.9%)	92 (20.4%)	0.704
Vaccinated at least once	115 (83.3%)	258 (82.7%)	373 (82.9%)	>0.999
Fully Vaccinated (at least 2 doses and 1 booster)	102 (73.9%)	213 (68.3%)	315 (70.0%)	0.265
Number of COVID-19 vaccine doses				0.495
- None	23 (16.7%)	54 (17.3%)	77 (17.1%)	
- One dose	1 (0.7%)	4 (1.3%)	5 (1.1%)	
- Two doses	12 (8.7%)	41 (13.1%)	53 (11.8%)	
- Two doses + 1 booster	71 (51.4%)	160 (51.3%)	231 (51.3%)	
- Two doses + 2 boosters	31 (22.5%)	53 (17.0%)	84 (18.7%)	

**Table 3:** Remdesivir patterns of use and timing. All figures are median (IQR) or N (%).

	Group A (Incidental COVID-19 Cases) (N = 138)	Group B (High-Risk Outpatients) (N = 312)	Total (N = 450)	*p*-Value
Duration of remdesivir regimen (days)				<0.001
- 1	0 (0.0%)	1 (0.3%)	1 (0.2%)	
- 2	1 (0.7%)	2 (0.6%)	3 (0.7%)	
- 3	68 (49.3%)	305 (97.8%)	373 (82.9%)	
- 4	7 (5.1%)	2 (0.6%)	9 (2.0%)	
- 5	59 (42.8%)	2 (0.6%)	61 (13.6%)	
- 6	2 (1.5%)	0 (0.0%)	2 (0.4%)	
- 7	1 (0.7%)	0 (0.0%)	1 (0.2%)	
Time from admission to remdesivir initiation (group A only)	4 (0, 7)	-	-	-
Time from first positive COVID-19 test to remdesivir initiation	1 (0, 1)	2 (1, 3)	2 (1, 3)	< 0.001

**Table 4:** Clinical Progression, rehospitalization and mortality.

	Group A (Incidental COVID-19 Cases) (N = 138)	Group B (High-Risk Outpatients) (N = 312)	Total (N = 450)	*p* Value
Clinical deterioration	8 (5.8%)	2 (0.6%)	10 (2.2%)	0.002
Admission to Intensive Care Unit	2 (1.4%)	0 (0.0%)	2 (0.4%)	0.033
Need for intubation	2 (1.4%)	0 (0.0%)	2 (0.4%)	0.033
Death within 30 days of admission (group A) or remdesivir initiation (group B)	25 (18.1%)	2 (0.6%)	27 (6.0%)	<0.001
New hospitalization within 30 days from discharge (group A) or completion of remdesivir treatment (group B)	0 (0.0%)	3 (1.0%)	3 (0.7)	>0.556
Reason for new hospitalization				
- Not related to COVID-19		3 (100.0%)	3 (100.0%)	
- Related to COVID-19		0 (0.0%)	0 (0.0%)	
Death up to 30 days from discharge (group A) or completion of remdesivir treatment (group B)	32 (23.2%)	2 (0.6%)	34 (7.6%)	< 0.001

**Table 5:** Renal and hepatic abnormalities.

	Group A (Incidental COVID-19 Cases) (N = 138)	Group B (High-Risk Outpatients) (N = 312)	Total (N = 450)	*p*-Value
Acute kidney injury (associated with remdesivir)				0.003
- Before or same day as Remdesivir Initiation	0 (0.0%)	1 (0.3%)	1 (0.2%)	
- More than 2 days after Remdesivir Stop	5 (3.6%)	0 (0.0%)	5 (1.1%)	
Hepatotoxicity (associated with remdesivir)				0.096
- Before or same day as Remdesivir Initiation	1 (0.7%)	1 (0.3%)	2 (0.4%)	
- During Remdesivir administration (up to 2 days after stop)	3 (2.2%)	1 (0.3%)	4 (0.9%)	
AST/ALT levels in Hepatotoxicity cases				0.033
3 to 5 xULN	4 (2.9%)	1 (0.3%)	5 (1.1%)	
>5 xULN	0 (0.0%)	1 (0.3%)	1 (0.2%)	

## Data Availability

The original contributions presented in this study are included in the article. Further inquiries can be directed to the corresponding author.
